# Neutrophil degranulation biomarkers characterize restrictive echocardiographic pattern with diastolic dysfunction in patients with diabetes

**DOI:** 10.1111/eci.13640

**Published:** 2021-06-24

**Authors:** Stefano Ministrini, Francesco Andreozzi, Fabrizio Montecucco, Silvia Minetti, Maria Bertolotto, Luca Liberale, Gaia Chiara Mannino, Elena Succurro, Velia Cassano, Sofia Miceli, Maria Perticone, Giorgio Sesti, Angela Sciacqua, Federico Carbone

**Affiliations:** ^1^ Internal Medicine, Angiology and Atherosclerosis Department of Medicine and Surgery Università degli Studi di Perugia Perugia Italy; ^2^ Center for Molecular Cardiology University of Zürich Schlieren Switzerland; ^3^ Department of Medical and Surgical Sciences University ‘Magna Græcia’ of Catanzaro Catanzaro Italy; ^4^ Department of Internal Medicine First Clinic of internal Medicine University of Genoa Genoa Italy; ^5^ IRCCS Ospedale Policlinico San Martino Genoa – Italian Cardiovascular Network Genoa Italy; ^6^ Department of Clinical and Molecular Medicine University of Rome‐Sapienza Rome Italy

**Keywords:** diabetic cardiomyopathy, echocardiography, neutrophils, resistin, type 2 diabetes mellitus

## Abstract

**Objective:**

To investigate the potential association between neutrophil degranulation and patterns of myocardial dysfunction in a cohort of patients with type 2 diabetes mellitus (T2DM).

**Background:**

Two distinct phenotypes of diabetic cardiomyopathy have been described: a restrictive phenotype with diastolic dysfunction (restrictive/DD) and a dilative phenotype with systolic dysfunction (dilative/SD). However, the underlying determinants of these two patterns are not yet recognized.

**Methods:**

In this single‐centre, observational, cross‐sectional study, 492 patients were recruited. Ultrasonographic measurements were performed by two experienced sonographers, blinded to the clinical data of the participants. Serum biomarkers of neutrophil degranulation were measured by enzyme‐linked immunosorbent sandwich assay (ELISA).

**Results:**

After adjustment for confounders, resistin, myeloperoxidase, matrix metalloproteinase 8 and matrix metalloproteinase 9/tissue inhibitor of metalloproteinases 1 complex were positively associated with the restrictive/DD pattern compared with the normal pattern. Similarly, MPO was positively associated with the dilative/SD pattern compared with the normal pattern, and resistin was negatively associated with the dilative/SD pattern compared with the restrictive/DD pattern.

**Conclusions:**

Neutrophil degranulation is associated with the restrictive/DD echocardiographic pattern in patients with T2DM, but not with the normal pattern and dilative/SD patterns. Neutrophils could have a pivotal role in the pathogenesis of myocardial dysfunction, and particularly diastolic dysfunction, in patients with T2DM.

## 1 INTRODUCTION

Heart failure is a common comorbidity in patients with diabetes mellitus (DM), with heavy drawbacks on their quality of life and life expectancy, and consequent relevant social costs.^1^ DM, and particularly type 2 DM (T2DM), is frequently associated with hypertension, obesity, lipid metabolism disorders and coronary artery disease; these factors could mediate the association between DM and heart failure. However, accumulating evidence highlights an additional disease‐specific role of DM on myocardial remodelling and dysfunction, leading to the definition of diabetic cardiomyopathy as a primary myocardial disease.^2^, ^3^ Two distinct phenotypes of diabetic cardiomyopathy have been described: a restrictive phenotype with diastolic dysfunction (restrictive/DD) and a dilative phenotype with systolic dysfunction (dilative/SD). These two phenotypes—once considered consecutive stages of the same condition—are now rather considered as independent entities with different pathogeneses and divergent natural course.^4^ The restrictive/DD pattern is characterized by a normal volume of the left ventricle, with a thickened and stiff wall. Enlarged cardiomyocytes with a preserved sarcomeric ultrastructure and increased deposition of extracellular matrix are the hallmark of this phenotype.^5^ Conversely, the dilative/SD pattern is characterized by an increased volume of the left ventricle. Cardiomyocytes are damaged with subversion of the sarcomeric structure and associated diffuse fibrosis. Both phenotypes are characterized by microvascular rarefaction and perivascular deposition of advanced glycation end products, more predominant in the dilative/SD phenotype.^4^, ^6^ Less is known about the role of innate immunity, and especially neutrophils, in the pathogenesis of diabetic cardiomyopathy and its subtypes.^7^ The aim of this study was to investigate the potential association between neutrophil degranulation—evaluated through a panel of validated serum biomarkers—and echocardiographic patterns of myocardial dysfunction in a cohort of patients with T2DM.

## 2 MATERIALS AND METHODS

### 2.1 Enrolled subjects and study design

The present study has a single‐centre, observational, cross‐sectional design enrolling Caucasian subjects participating in the CATAnzaro MEtabolic RIsk factors (CATAMERI) study.^8^ Inclusion criteria were an established diagnosis of T2DM according to the American Diabetes Association diagnostic criteria^9^ and age >18 years. Exclusion criteria were as follows: previous myocardial infarction or previous coronary revascularization; known coronary artery disease, valvular abnormality or hypertensive heart disease; and the presence of end‐stage renal disease, chronic gastrointestinal diseases associated with malabsorption, chronic pancreatitis, history of any malignant disease, self‐reporting alcohol consumption >20 g/d, positivity for antibodies to hepatitis C virus or hepatitis B surface antigen. The protocol (approval code: 2012.63) was approved by the Local Ethics Committee of Catanzaro (Comitato Etico Azienda Ospedaliera ‘Mater Domini’), and written informed consent was obtained from each subject before commencing the study, in accordance with the principles of the Declaration of Helsinki.

### 2.2 History, clinical parameters and anthropometric measures

Personal history, family history and current pharmacological treatments were collected using a standardized questionnaire. The anthropometric assessment was illustrated in the Supplementary Material.

### 2.3 Biochemical parameters

Venous blood was withdrawn in the morning after at least 8‐hour fasting. Blood specimens were immediately centrifuged to separate sera and then frozen at −80°C for storage. Alongside serum levels of C‐reactive protein (CRP), considered a generic biomarker of inflammation in several classes of disease including metabolic ones,^10^, ^11^, ^12^, ^13^ we assessed a validated panel of biomarkers providing surrogate evidence of neutrophil degranulation: resistin, myeloperoxidase (MPO), matrix metalloproteinase (MMP) 8 and 9, tissue inhibitor of metalloproteinase (TIMP) 1 and 2 and MMP‐9/TIMP‐1 complex.^14^, ^15^, ^16^, ^17^ All those biomarkers were measured by enzyme‐linked immunosorbent sandwich assay with double‐automated colorimetric reading, following the manufacturer's instructions (R&D Systems; Minneapolis, MN). The lowest detection thresholds were as follows: 15.625 pg/mL for CRP; 31.25 pg/mL for resistin, MMP‐9, TIMP‐1 and TIMP‐2; 46.88 pg/mL for MMP‐9/TIMP‐1 complex; and 62.5 pg/mL for MPO and MMP‐8. Intra‐ and interassay coefficient of variation was below 8% for all markers, as previously described.^16^, ^17^ Glucose, triglycerides, and total and high‐density lipoprotein (HDL) cholesterol concentrations were measured by enzymatic methods (Roche, Basel, Switzerland); plasma insulin concentration was measured with a chemiluminescence‐based assay (Immulite^®^; Siemens Healthcare GmbH, Erlangen, Germany); white blood cell (WBC) count and neutrophils were measured using an automated particle counter (ADVIA 120/2120 Haematology System; Siemens Healthcare Diagnostics); and HbA1c was determined with high‐performance liquid chromatography using a National Glycohemoglobin Standardization Program (NGSP)‐certified automated analyser (Adams HA‐8160 HbA1C Analyzer, Menarini).

### 2.4 Echocardiographic analysis

All measurements were performed by two experienced sonographers, blinded to the clinical data of the participants. Echocardiographic measures were collected according to literature, as reported in the Supplementary Material.

The definition and grading of left ventricular (LV) diastolic dysfunction were adapted from the recommendations of the EACVI,^18^ as follows:

- No dysfunction (Grade 0): E/A ratio between 0.8 and 1.9 + E/e’ ratio <8.0 or left atrial diameter (LAD) ≤4.2 cm, if E/e’ ratio between 8.0 and 15.0;
- Mild dysfunction (Grade 1): E/A ratio <0.8;
- Moderate dysfunction (Grade 2): E/A ratio between 0.8 and 1.9 + E/e’ ratio >15 or LAD >4.2 cm, if E/e’ ratio between 8.0 and 15.0;
- Severe dysfunction (Grade 3): E/A ratio ≥2.0.

The definition and grading of LV systolic dysfunction were based on values of LV ejection fraction (LVEF) measured through Simpson's biplane formula^19^ and the tissue Doppler analysis,^20^ as follows:

- No dysfunction (Grade 0): LVEF ≥55% and LV s’ ≥0.075 m/s;
- Mild dysfunction (Grade 1): LVEF between 40% and 55% or LV s’ between 0.03 and 0.075 m/s;
- Severe dysfunction (Grade 2): LVEF <40% or LV s’ <0.03 m/s;

The presence and severity of LV dilation were assessed according to the indexed LV end‐diastolic volume (LVEDDi)^21^

- No dilation (Grade 0): ≤75 mL/m^2^;
- Mild dilation (Grade 1): 76‐86 mL/m^2^;
- Moderate dilation (Grade 2): 87‐96 mL/m^2^;
- Severe dilation (Grade 3): ≥97 mL/m^2^.

The presence and severity of LV hypertrophy were assessed according to the indexed LV mass (LVMi) ^21^

- No hypertrophy (Grade 0): ≤115 g/m^2^
- Mild hypertrophy (Grade 1): 116‐131 g/m^2^
- Moderate hypertrophy (Grade 2): 132‐148 g/m^2^
- Severe hypertrophy (Grade 3): ≥149 g/m^2^

Echocardiographic patterns in patients with diabetes mellitus were defined according to Seferović and colleagues^4^

- Dilative pattern with systolic dysfunction (dilative/SD): the presence of any LV systolic dysfunction and/or severe LV dilation;
- Restrictive pattern with diastolic dysfunction (restrictive/DD): the presence of any diastolic dysfunction in a subject not meeting the criteria of dilative/ SD;
- Normal pattern: the absence of criteria for either dilative/ SD or restrictive/ DD.

### 2.5 Endpoint adjudication and power study calculation

The primary endpoint of the study was the detection of whether surrogate biomarkers of neutrophil degranulation correlate with at least two echocardiographic patterns of myocardial dysfunction. The secondary endpoint was a significant correlation between the over‐mentioned biomarkers and the ultrasonographic parameters of myocardial dysfunction.

The adequacy of sample size was assessed assuming a 60% prevalence of subclinical myocardial dysfunction in patients with T2DM, with an equal prevalence of dilative/SD and restrictive/DD pattern.^22^ Based on previous studies in patients with heart failure, the minimum clinically relevant difference for resistin was calculated as 0.93 ng/mL, with a SD for difference of 1.64 ng/mL.^23^ As a result, a sample size of 243 patients is considered sufficient to reach the desired power of 0.95.

### 2.6 Statistical analysis

Continuous variables are expressed as median and interquartile range (IQR) or mean ± standard deviation (SD) according to their distribution assessed with the Kolmogorov‐Smirnov test. Categorical variables are instead presented as number (%). A probability of type 1 error <0.05 is considered significant under the assumption of null hypothesis. Overall significance of differences between groups was assessed by the Kruskal‐Wallis test and the chi‐square test for continuous and categorical parameters, respectively. The significance of pairwise differences was assessed through the Mann‐Whitney *U* test or univariate logistic regression, after log transformation of non‐normally distributed variables. The correction for potential confounders was performed using multiple logistic regression analysis. The analysis was performed using the SPSS software package 23.0 (IBM Inc.).

## 3 RESULTS

### 3.1 Clinical and biochemical variables and echocardiographic assessment of the study cohort

Demographic, physical and laboratory characteristics of the enrolled population are reported in Table 1. The enrolled population comprised 492 subjects, with a prevalence of male subjects (64.0%) in their late adulthood (median age: 61 years), who received a relatively recent diagnosis of T2DM (median duration of disease: 4 years). Most of the female subjects were in post‐menopausal age (64%). Enrolled patients were overall obese (median BMI: 30.5 kg/m^2^) with a good glycometabolic control (median HbA1c: 6.9%). As reported in Table S1, the majority of patients had associated hypertension (88.1%) and/or metabolic syndrome (77.7%). Three hundred and sixty subjects were on pharmacological treatment for hypertension, with inhibitors of renin‐angiotensin‐aldosterone system being the most prescribed class of drugs (308 subjects). Most of the patients showed a low‐grade inflammatory profile (median CRP level: 3.2 mg/L).

**TABLE 1:** Demographic, physical and laboratory characteristics of enrolled patients

Variables	
Age (y)	61 [53‐69]
Duration of diabetes (y)	4 [0‐12]
Sex (females)	178 (36.0)
Post‐menopausal status	114 (64.0)
Duration of menopause (y)	17 [8‐25]
Body weight (kg)	82 [73‐95]
BMI (kg/m^2^)	30.5 [27.0‐34.5]
Waist circumference (cm)	105 [98‐115]
Systolic blood pressure (mm Hg)	135 [120‐148]
Diastolic blood pressure (mm Hg)	80 [70‐90]
White blood cells × 10^3^/mL	7.4 [6.2‐8.8]
Neutrophils 10^3^/mL	4.1 [3.3‐5.1]
Fasting blood glucose (mg/dL)	133 [113‐168]
Fasting insulin (UI/mL)	14.0 [9.3‐22.0]
HOMA‐IR	4.7 [2.9‐7.8]
HOMA‐β%	78.5 [42.3‐136.0]
HbA1c (%)	6.9 [6.2‐8.2]
Total cholesterol (mg/dL)	184 [152‐218]
Triglycerides (mg/dL)	137 [103‐188]
HDL‐cholesterol (mg/dL)	44 [37‐53]
LDL‐cholesterol (mg/dL)	111 [83‐140]
CRP (mg/L)	3.2 [1.3‐5.5]
Resistin (ng/mL)	28.9 [17.3‐47.6]
MPO (ng/mL)	298.3 [158.5‐588.4]
MMP‐8 (ng/mL)	21.5 [10.5‐41.3]
MMP‐9 (ng/mL)	465.5 [268.5‐727.9]
MMP‐9/TIMP‐1 complex (ng/mL)	8.8 [4.2‐17.9]
TIMP‐1 (ng/mL)	177.5 [137.9‐216.4]
TIMP‐2 (ng/mL)	74.0 [59.8‐93.3]

Note

Continuous variables are presented as median [IQR], whereas categorical ones, as absolute count (%).

Abbreviations: BMI, body mass index; CRP, C‐reactive protein; HbA1c, glycated haemoglobin; HDL, high‐density lipoprotein; HOMA‐IR, homeostatic model of assessment—insulin resistance; HOMA‐β%, homeostatic model of assessment percentage of beta‐cell function; LDL, low‐density lipoprotein; MMP: matrix metalloproteinase; MPO: myeloperoxidase; TIMP: tissue inhibitor of metalloproteinase.

**FIGURE 1:**
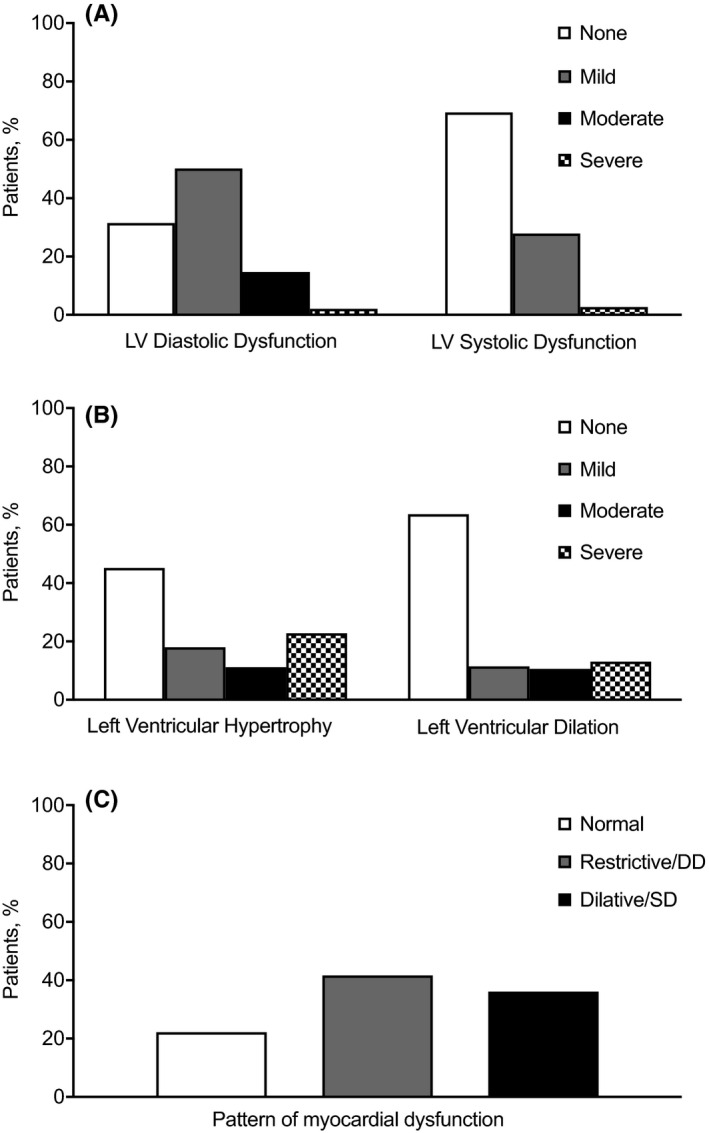
Distribution of echocardiographic patterns across the study cohort. Echocardiographic assessment of study patients according to the presence of left ventricular (LV) diastolic/systolic dysfunction (A) and LV hypertrophy/dilation (B) categorized as none, mild, moderate or severe. We also report the distribution of patterns of myocardial dysfunction as normal, restrictive/diastolic dysfunction (DD) and dilative/systolic dysfunction (SD) (C)

Complete echocardiographic parameters were available for 451 patients. Median values and IQR of parameters analysed in the present study are reported in Table S2. LV diastolic dysfunction was diagnosed in 63.8% of subjects, and LV systolic dysfunction, in 30.6% (Figure 1A); and LV hypertrophy was diagnosed in 52.3% of subjects, and LV dilation, in 35.7% (Figure 1B). A restrictive pattern with diastolic dysfunction was observed in 177 subjects (35.8%), whereas a dilative pattern with systolic dysfunction was observed in 163 subjects (36.1%) (Figure 1C).

### 3.2 Neutrophil‐related inflammatory biomarkers correlate with parameters of left ventricular dysfunction

As expected, inflammatory parameters belonging to neutrophil degranulation were positively correlated with neutrophils and white blood cell counts, with CRP levels and with each other (Table S3) It is noteworthy that CRP also positively correlated with body weight, the presence of metabolic syndrome, the markers of insulin resistance and LDL‐cholesterol (Table S4). As regards the parameters of LV dysfunction, serum levels of CRP, resistin, MPO, MMP‐8 and MMP‐9 and TIMP‐1 positively correlated with the E/e’ ratio; among them, resistin, MPO and TIMP‐1 also positively correlated with the degree of diastolic dysfunction. MPO and TIMP‐1 were also negatively correlated with the LV s’ (Table 2). As regards the parameters of LV remodelling, resistin positively correlated with interventricular septum (IVS) thickness and negatively correlated with the LV diameters and volumes. Similarly, MMP‐9 positively correlated with the LV posterior wall (LVPW) thickness and negatively correlated with the LVEDVi (Table 2).

**TABLE 2:** Correlations between neutrophil activity biomarkers and left ventricular function

LV function	Diastolic								Systolic					
	Left atrial diameter		E/A ratio		E/e’ ratio		Grade of dysfunction		LV ejection fraction		LV s’		Grade of dysfunction	
	*ρ*	*P*‐value	*ρ*	*P*‐value	*ρ*	*P*‐value	*ρ*	*P*‐value	*ρ*	*P*‐value	*ρ*	*P*‐value	*ρ*	*P*‐value
CRP	−0.014	0.779	0.037	0.476	**0.138**	**0.004**	0.061	0.234	0.040	0.414	0.012	0.810	0.028	0.569
Resistin	−0.005	0.916	−0.080	0.089	**0.195**	**<0.001**	**0.167**	**<0.001**	0.089	0.052	−0.036	0.429	−0.041	0.366
MPO	−0.016	0.725	−0.028	0.551	**0.303**	**<0.001**	**0.130**	**0.006**	**0.094**	**0.041**	**−0.097**	**0.033**	0.026	0.563
MMP‐8	−0.050	0.277	−0.026	0.588	**0.165**	**<0.001**	0.077	0.107	0.022	0.636	−0.053	0.240	−0.017	0.703
MMP‐9	−0.023	0.617	0.035	0.455	**0.289**	**<0.001**	**0.096**	**0.044**	0.022	0.628	−0.069	0.126	0.043	0.340
MMP‐9/TIMP‐1 complex	−0.035	0.448	−0.091	0.055	−0.023	0.611	0.093	0.051	−0.009	0.849	−0.019	0.668	0.013	0.775
TIMP‐1	0.047	0.308	−0.078	0.098	**0.256**	**<0.001**	**0.160**	**0.001**	0.053	0.248	**−0.115**	**0.011**	0.046	0.313
TIMP‐2	−0.015	0.739	−0.058	0.221	−0.008	0.862	0.056	0.238	0.004	0.924	−0.049	0.282	0.019	0.677

LV remodelling	Volume								Wall thickness					
	LV end‐diastolic diameter		LV end‐systolic diameter		LV end‐diastolic volume indexed		LV end‐systolic volume		IVS thickness		LVPW thickness		LVMi	
	*ρ*	*P*‐value	*ρ*	*P*‐value	*ρ*	*P*‐value	*ρ*	*P*‐value	*ρ*	*P*‐value	*ρ*	*P*‐value	*ρ*	*P*‐value
CRP	−0.014	0.774	−0.031	0.523	−0.051	0.302	−0.063	0.208	−0.079	0.107	−0.029	0.150	−0.061	0.218
Resistin	**−0.094**	**0.038**	**−0.101**	**0.027**	**−0.099**	**0.033**	**−0.107**	**0.021**	**0.408**	**0.025**	0.049	0.286	0.026	0.583
MPO	−0.060	0.189	−0.084	0.067	−0.067	0.146	**−0.097**	**0.037**	−0.006	0.892	**0.114**	**0.013**	0.054	0.247
MMP‐8	−0.062	0.177	−0.054	0.239	−0.067	0.146	−0.097	0.037	0.011	0.809	0.014	0.767	0.045	0.332
MMP‐9	**−0.100**	**0.028**	−0.081	0.078	−0.083	0.073	−0.073	0.119	−0.038	0.409	0.040	0.380	−0.017	0.712
MMP‐9/TIMP‐1 complex	−0.046	0.318	−0.019	0.682	−0.073	0.117	−0.044	0.338	0.043	0.347	0.023	0.621	−0.056	0.770
TIMP‐1	−0.063	0.169	−0.067	0.142	−0.043	0.356	−0.060	0.200	0.035	0.444	0.012	0.802	−0.063	0.743
TIMP‐2	−0.050	0.273	−0.044	0.337	−0.053	0.249	−0.041	0.374	0.013	0.782	**−0.109**	**0.017**	−0.042	0.373

Note

Comparisons were performed by Spearman's rank correlation

Values in bold are statistically significant (*p* < 0.05).

Abbreviations: CRP, C‐reactive protein; LV, left ventricle; MMP, matrix metalloproteinase; MPO, myeloperoxidase; TIMP, tissue inhibitor of metalloproteinase.

### 3.3 Neutrophil‐related inflammatory biomarkers are independently associated with patterns of myocardial dysfunction

**FIGURE 2:**
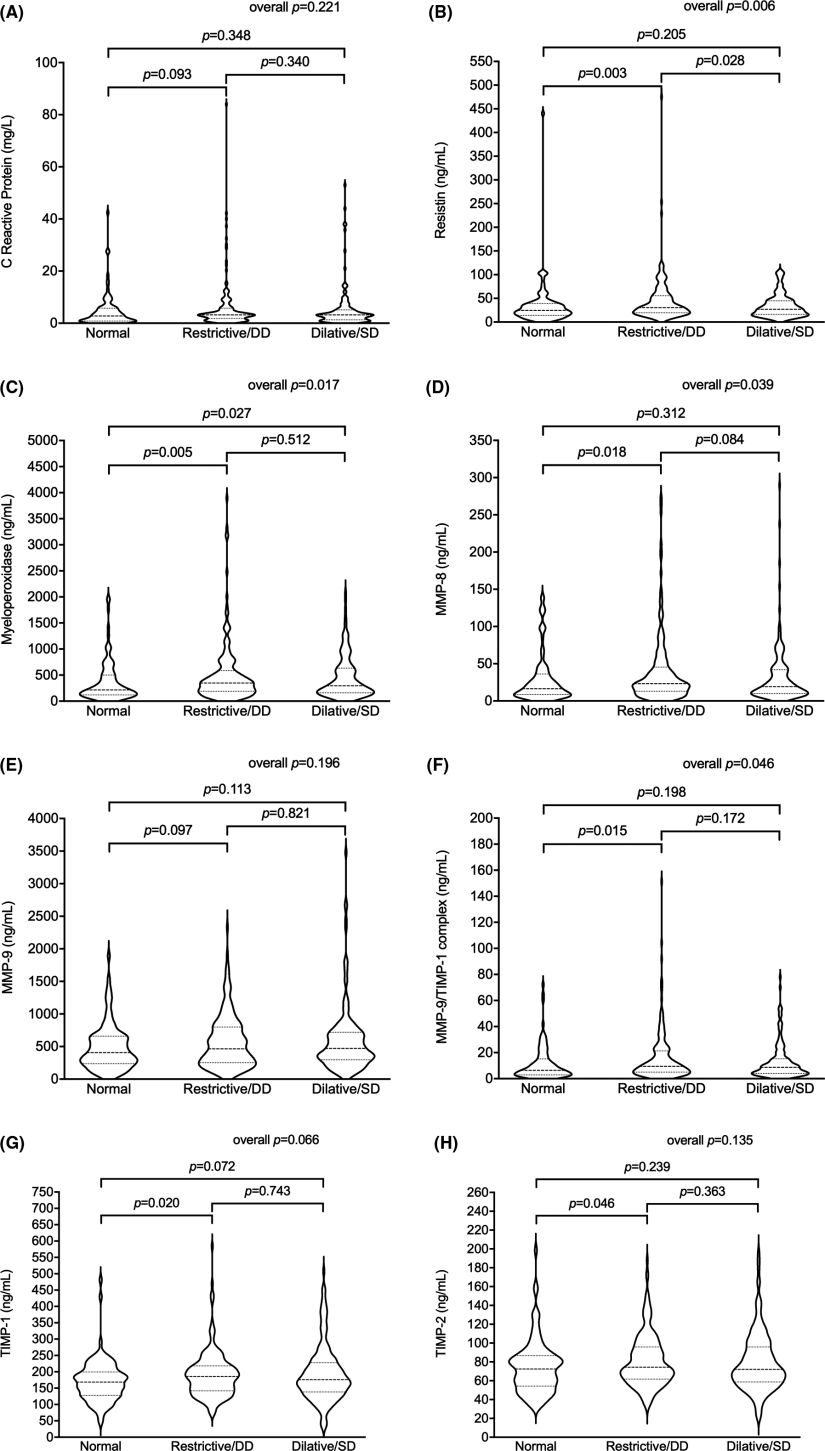
High circulating levels of neutrophil‐related biomarkers are associated with myocardial dysfunction. As compared to C‐reactive protein (CRP) (A), high circulating levels of resistin characterize both restrictive/diastolic dysfunction (DD) and dilative/systolic dysfunction patterns (B). More generally, restrictive/DD pattern, in particular, is characterized by the increase in circulating levels of myeloperoxidase (MPO) (C), matrix metalloproteinase (MMP)‐8 (D), MMP‐9 (E), MMP‐9/tissue inhibitor of metalloproteinase (TIMP)‐1 (F), TIMP‐1 (G) and TIMP‐2 (H)

**FIGURE 3:**
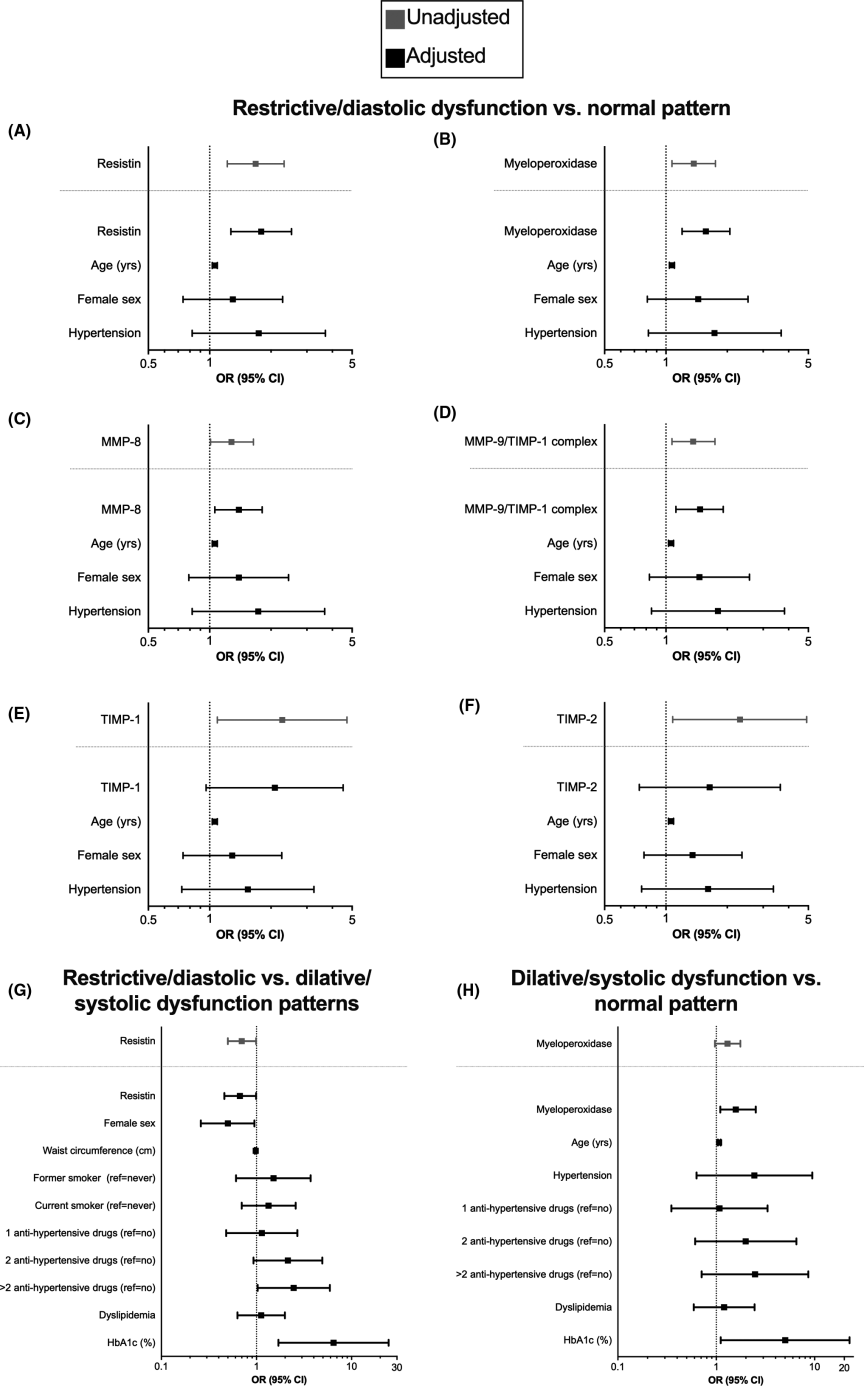
Neutrophil‐related cytokines are independently associated with the development of left ventricular dysfunction. Restrictive/diastolic dysfunction pattern is independently associated with high circulating levels of neutrophil‐related cytokines: resistin (A), myeloperoxidase (MPO) (B), matrix metalloproteinase (MMP)‐8 (C), and MMP‐9/tissue inhibitor of metalloproteinase (TIMP)‐1 (D), TIMP‐1 (E) and TIMP‐2 (F). Adjusted analyses confirmed these finding except for TIMP‐1 and TIMP‐2. Resistin was further confirmed as associated with the restrictive pattern as compared to the dilative one (G), while high circulating levels of MPO also characterize the dilative pattern (H)

Comparing the average values of neutrophil degranulation biomarkers among echocardiographic patterns (normal, restrictive with diastolic dysfunction and dilative with systolic dysfunction), a significant difference between normal and restrictive patterns was observed for resistin, MPO, MMP‐8, MMP‐9/TIMP‐1 complex, TIMP‐1 and TIMP‐2 (Figure 2). A significant difference in MPO values was also observed between normal and dilative patterns, whereas serum resistin levels significantly differed in restrictive and dilative patterns. Conversely, CRP did not differ across the different patterns of myocardial dysfunction. When compared to clinical, anthropometric and biochemical parameters, significant differences among the echocardiographic patterns were observed (Table S5). Those variables were then considered as potential confounders and included in logistic regression models (Figure S1 and Table S6). After adjustments, resistin, MPO, MMP‐8 and MMP‐9/TIMP‐1 complex confirmed their independent association with the restrictive pattern compared with the normal pattern (Figure 3 and Tables S6 and S7). Similarly, MPO was positively associated with the dilative pattern compared with the normal pattern, and resistin was negatively associated with the dilative pattern compared with the restrictive pattern (Figure 3 and Tables S8 and S9).

## 4 DISCUSSION

The main finding of the present study is the association between neutrophil degranulation, evaluated through surrogate serum biomarkers, and the restrictive/DD pattern of myocardial dysfunction, evaluated through echocardiography, in patients with T2DM. This association is confirmed also after adjustment for potential confounders. Specifically, measured biomarkers are significantly higher in patients with restrictive/DD pattern than in those with normal pattern. Such difference has not been observed between patients with dilative/SD pattern and normal pattern, with the exception of MPO, showing significantly elevated values also in the dilative/SD pattern group. Notably, resistin values were significantly higher in the restrictive/DD pattern group than in the dilative/SD pattern group, whereas CRP failed to show any difference across the patterns of myocardial dysfunction. Overall, these findings might suggest some specific role for neutrophil degranulation in the pathophysiology of diabetic cardiomyopathy. This would not be surprising as the involvement of leucocyte in diabetic cardiomyopathy has been well established.^7^ However, less is known about the role of neutrophils.^24^, ^25^ Actually, they are more prone to oxidative burst and secretion of extracellular traps, whereas phagocytic and chemotactic activity is reduced in patients with DM.^26^, ^27^, ^28^ With regard to myocardial function, neutrophil degranulation has been associated with increased intraventricular diastolic pressure in patients with hypertensive heart disease^29^ and severe chronic kidney disease,^30^ but not yet in diabetic patients. Well established is instead the association between MMPs and LVDD in animal models.^31^

Equally interesting is the evidence about the potential involvement of resistin in diabetic cardiomyopathy. Traditionally known as is cysteine‐rich adipokine with downregulating effect of insulin signalling,^32^ resistin is highly expressed in mononuclear cells and stored in the primary granules of neutrophils.^33^ It has been supposed that resistin might sustain the chronic low‐grade inflammation characterizing the metabolic disorders,^34^ but clear evidence is lacking.^17^, ^35^ Indeed, only one single study has so far reported a positive association between resistin and myocardial diastolic dysfunction.^36^

Instead, why MPO, MMP‐8, TIMP‐1, ‐2, and MMP‐9/TIMP‐1 as well are significantly associated with restrictive/DD, whereas only MPO is associated with dilative/SD still remains a controversial point. Although speculative, the development of restrictive/DD pattern can be assumed as a more complex process involving the deposition of extracellular matrix. This could be linked to a different neutrophil activation/degranulation pattern. In particular, MMP‐8, and MMP‐9—when balanced with TIMPs—exert pro‐fibrotic effects.^37^ Unfortunately, the study design of the primary cohort did not plan to collect neutrophils and we cannot perform any ex vivo experiment on neutrophil function. This should be considered a leading shortcoming of the present study. Beyond neutrophil degranulation biomarkers, we should also acknowledge that other determinants of echocardiographic patterns should be accounted for such as demographic, clinical and biochemical factors; namely, the presence of any abnormal pattern (both restrictive/DD and dilative/SD) was characterized by older age and the presence of hypertension.^1^ The restrictive/DD pattern was further characterized by the female sex, whereas the dilative/SD pattern was characterized by abdominal obesity, active smoking, higher severity of hypertension (estimated through the number of anti‐hypertensive drugs), dyslipidaemia and worse glycometabolic control. Since the aim of the study was to investigate a correlation between serum biomarkers of neutrophil degranulation and myocardial function in patients with T2DM, subjects were not screened for the presence of coronary artery disease before enrolment, and neither were they retrospectively excluded on the basis of diagnostic tests performed afterwards. This could represent a limitation when attempting to implementing these results in the setting of diabetic cardiomyopathy. Furthermore, the definition of diastolic dysfunction was simplified in order to ease data collection and analysis, since the study does not aim at establishing an accurate diagnosis, rather detecting an association between a panel of biomarkers and an echocardiographic pattern. Notwithstanding this limitation, the prevalence of diastolic dysfunction reported in this paper is consistent with the one observed in previous studies.^22^

As an additional limitation, we should acknowledge that this study is composed of patients with a relatively recent diagnosis of diabetes. This may somewhat limit the generalization of results, but it may also point out how neutrophil degranulation contributes earlier to the development of diabetic cardiomyopathy.

Finally, the prevalence of hypertension, obesity and metabolic syndrome was quite homogeneous among the three groups. Although model adjustments for these potential confounders were adopted, a relevant contribution of these comorbidities to myocardial dysfunction cannot be excluded. In particular, the adjustment for hypertension only concerns the presence of disease and the number of anti‐hypertensive drugs, but not the duration of hypertension. The latter is often difficult to estimate as it often occurs asymptomatically but undoubtedly represents a missing confounder that should be acknowledged as a further study limitation.

In conclusion, the results of the present study suggest that neutrophil degranulation characterizes the restrictive/DD echocardiographic pattern in patients with T2DM, compared with the normal pattern and the dilative/SD pattern as well. This result is consistent with previous observations in animal models but not in human beings. Future studies are needed to assess whether serum biomarkers could help in stratifying the risk of myocardial dysfunction in subjects with T2DM and normal myocardial function, as well as investigating the potential pharmacological strategies targeting neutrophil activity (eg inhibitors of MMPs) to prevent the progression of myocardial dysfunction to overt heart failure.

## CONFLICT OF INTEREST

The authors declare they have no conflict of interest.

## Supporting information

Supplementary Material
