# Supplementary material for: Neutrophil degranulation biomarkers characterize restrictive echocardiographic pattern with diastolic dysfunction in patients with diabetes
Source: Eur J Clin Invest. 2021 Jun 24;51(12):e13640. doi: 10.1111/eci.13640 (PMC9286613; doi:10.1111/eci.13640)
Supplement: Supplementary file 1 — Supplementary Material [file ECI-51-0-s001.docx]

**Supplementary Material**

We would also include the following paragraph belonging to Method Section and Tables as Supplementary Material as they provided additional data and completed information.

# **History, clinical parameters and anthropometric measures**

Weight and height were measured by means of a scale; the BMI was calculated as

$BMI ({kg}/{m^{2})}=\frac{weight (kg)}{{height (m)}^{2}}$.

Body surface area was estimated with the DuBois formula ([1](#_ENREF_1))

$BSA \left( m^{2} \right)=0.020247\times{height (m)}^{0.725}\times{weight (kg)}^{0.425}$.

Waist circumference was measured by means of a tape placed midway between the inferior rib margin and the anterior superior iliac crest, with the patient standing. Blood pressure was measured using a standard sphygmomanometer in the sitting position, as the average of the last two of three consecutive measurements obtained at 3 min intervals. Measurements were performed in the morning, with the patient fasting and sitting in a comfortable environment. An average of three consecutive measurements was considered. The Homeostasis Model Assessment of Insulin Resistance (HOMA-IR) was calculated as

$HOMA-IR=\frac{fasting glucose \left( {mmol}/{mL} \right)\times fasting insulin ({\mu IU}/{mL})}{405}$;

Homeostasis Model Assessment of β-cell function (HOMA-β%) was calculated as:

$HOMA-\beta=20\times\frac{fasting insulin ({\mu IU}/{mL})}{fasting glucose ({mmol}/{mL})}-3.5$.

Metabolic syndrome was defined, according to the National Cholesterol Education Panel – Adult Treatment Panel III (NCEP-ATPIII) criteria ([2](#_ENREF_2)), as the presence of at least 3 of the following criteria: waist circumference ≥88 cm for females and ≥102 cm for males; triglycerides ≥150 mg/dL, high-density lipoprotein cholesterol <50 mg/dL for females and <40 mg/dL for males; systolic blood pressure ≥135/85 mmHg or ongoing anti-hypertensive treatment; fasting blood glucose ≥110 mg/dL or established diagnosis of diabetes mellitus.

# **Echocardiograpic analysis**

Left atrial diameter (LAD) was measured in apical two-chamber view at the end of systole, excluding the pulmonary veins. The LAD was measured as the maximum distance between the mitral valve plane and the roof of the left atrium ([3](#_ENREF_3)). LAD values above the 90^th^ percentile (4.2 cm) were considered abnormal ([4](#_ENREF_4)).

Left ventricular end-diastolic and end-systolic diameters (LVEDD and LVESD) were measured using M-mode from the parasternal long‐axis view at the level of the mitral valve leaflet tips. Left ventricular end-diastolic and end-systolic volumes (LVEDV and LVESV) were calculated using the biplane Simpson’s method from an apical four-chamber view. Left ventricular ejection fraction (LVEF) was calculated with the following formula:

$LVEF=(\frac{LVEDV-LVESV}{LVEDV})\times100$ ([5](#_ENREF_5)).

Interventricular septum thickness (IVST) and left ventricular posterior wall thickness (LVPWT) were measured from the parasternal long‐axis view at the level of the mitral valve leaflet tips, at the end of diastole according to the recommendations of the European association of Cardiovascular Imaging (EACVI). Left ventricular mass (LVM) was estimated as:

$$LVM (g)=1.04\times\left[ \left( IVST+LVPWT+LVEDD \right)^{3}-{LVEDD}^{3} \right]+0.6$$

and indexed (LVMi) to BSA ([6](#_ENREF_6)). Trans-mitral flow velocity was measured using pulse wave Doppler, with the sample volume placed at the tip of the mitral valve from an apical four-chamber view. Early (E) and late (A) left ventricular diastolic inflow velocities were measured and expressed as the E/A ratio ([7](#_ENREF_7)). Tissue Doppler Imaging (TDI) was employed to measure peak tissue velocity at the level of the mitral annulus during early diastole (e′) and systole (s’). Measures were recorded from an apical four-chamber view, with the sample volume placed at the septal and lateral corners of the mitral annulus ([8](#_ENREF_8)).

**References**

1. Sawyer M, Ratain MJ. Body surface area as a determinant of pharmacokinetics and drug dosing. Invest New Drugs 2001;19:171-7.

2. Expert Panel on Detection E, Treatment of High Blood Cholesterol in A. Executive Summary of The Third Report of The National Cholesterol Education Program (NCEP) Expert Panel on Detection, Evaluation, And Treatment of High Blood Cholesterol In Adults (Adult Treatment Panel III). JAMA 2001;285:2486-97.

3. Larsen CM VBCaSM. Principles of Measuring Chamber Size, Volume and Hemodynamic Assessment of the Heart. In: Springer, editor Echocardiography, 2018:111-28.

4. Stolzmann P, Scheffel H, Leschka S et al. Reference values for quantitative left ventricular and left atrial measurements in cardiac computed tomography. Eur Radiol 2008;18:1625-34.

5. Lang RM, Bierig M, Devereux RB et al. Recommendations for chamber quantification: a report from the American Society of Echocardiography's Guidelines and Standards Committee and the Chamber Quantification Writing Group, developed in conjunction with the European Association of Echocardiography, a branch of the European Society of Cardiology. J Am Soc Echocardiogr 2005;18:1440-63.

6. Marwick TH, Gillebert TC, Aurigemma G et al. Recommendations on the use of echocardiography in adult hypertension: a report from the European Association of Cardiovascular Imaging (EACVI) and the American Society of Echocardiography (ASE)dagger. Eur Heart J Cardiovasc Imaging 2015;16:577-605.

7. Nagueh SF, Smiseth OA, Appleton CP et al. Recommendations for the Evaluation of Left Ventricular Diastolic Function by Echocardiography: An Update from the American Society of Echocardiography and the European Association of Cardiovascular Imaging. Eur Heart J Cardiovasc Imaging 2016;17:1321-1360.

8. Ali SI, Li Y, Adam M, Xie M. Evaluation of Left Ventricular Systolic Function and Mass in Primary Hypertensive Patients by Echocardiography. J Ultrasound Med 2019;38:39-49.

# **Supplementary Table 1. Comorbidities and pharmacological therapies.**

|  | Number (%) |
| --- | --- |
| Smoking habit |  |
| Non-smokers | 232 (47.0) |
| Former smokers | 87 (17.6) |
| Current smokers | 173 (35.0) |
| Family history |  |
| Type 2 ^*^DM | 277 (56.1) |
| Hypertension | 284 (57.5) |
| Coronary Artery Disease | 192 (38.9) |
| Comorbidities |  |
| Hypertension | 435 (88.1) |
| Dyslipidemia | 204 (41.3) |
| Metabolic Syndrome | 384 (77.7) |
| Antidiabetic treatment |  |
| Diet and lifestyle intervention | 143 (28.9) |
| Biguanides | 187 (37.9) |
| Sulfonylureas | 73 (14.8) |
| Incretins | 11 (2.4) |
| Long-acting insulin | 44 (8.9) |
| Short-acting insulin | 57 (11.5) |
| SGLT-2 inhibitor | 1 (0.2) |
| Other | 8 (1.7) |
| Anti-hypertensive treatment |  |
| ^†^ACE-inhibitors/^‡^ARBs | 308 (67.0 |
| Diuretics | 169 (34.2) |
| Ca^2+^ antagonists | 103 (20.9) |
| β-blockers | 131 (26.5) |
| α-blockers | 16 (3.2) |
| Nitrates | 31 (6.3) |
| Number of anti-hypertensive drugs |  |
| 0 | 101 (20.4) |
| 1 | 116 (23.5) |
| 2 | 126 (25.5) |
| 3 or more | 118 (24.4) |
| Lipid lowering drugs |  |
| No/diet, n (%) | 257 (52.0) |
| Statin, n (%) | 197 (39.9) |
| Fibrate, n (%) | 5 (1.0) |
| Statin + fibrate, n (%) | 2 (0.4) |

^*^DM: diabetes mellitus; ^†^ACE: angiotensin converting enzyme; ^‡^ARBs: angiotensin II receptor blockers.

# **Supplementary Table 2. Echocardiographic parameters.**

| **Parameter** |  |
| --- | --- |
| **Left ventricular wall thickness** |  |
| Interventricular septum thickness, cm | 1.19 [1.08-1.28] |
| Left ventricular posterior wall thickness, cm | 0.94 [0.85-1.06] |
| Left ventricular mass indexed, g/m^2^ | 118 [98-146] |
| Relative Wall Thickness | 0.37 [0.33-0.42] |
|  |  |
| **Left ventricular volume** |  |
| Left ventricular end-diastolic diameter, cm | 5.07 [4.68-5.46] |
| Left ventricular end-systolic diameter, cm | 3.31 [3.00-3.71] |
| Left ventricular end-diastolic volume indexed, mL/m^2^ | 68.20 [54.57-82.34] |
| Left ventricular end-systolic volume indexed, mL/m^2^ | 19.20 [13.96-26.69] |
|  |  |
| **Left ventricular diastolic function** |  |
| Left atrial diameter, cm | 4.03 [3.69-4.40] |
| E/A ratio | 0.80 [0.70-0.91] |
| E/e’ ratio | 8.33 [6.85-10.00] |
|  |  |
| **Left ventricular systolic function** |  |
| Left ventricular ejection fraction, % | 63 [58-67] |
| Left ventricular s’, m/s | 0.09 [0.08-0.11] |

Continuous variables are presented and median [IQR].

# **Supplementary Table 3. Correlations among neutrophils activation biomarkers.**

|  |  | ^†^CRP | Resistin | ^‡^MPO | ^§^MMP-8 | MMP-9 | MMP-9/^\|\|^TIMP-1 complex | TIMP-1 | TIMP-2 |
| --- | --- | --- | --- | --- | --- | --- | --- | --- | --- |
| ^*^WBC | *ρ* | **0.293** | **0.239** | **0.188** | **0.246** | **0.361** | **0.335** | **0.213** | 0.034 |
|  | *p*-value | **<0.001** | **<0.001** | **<0.001** | **<0.001** | **<0.001** | **<0.001** | **<0.001** | 0.456 |
| Neutrophils | *ρ* | **0.380** | **0.236** | **0.203** | **0.150** | **0.259** | **0.237** | **0.222** | 0.104 |
|  | *p*-value | **<0.001** | **0.001** | **0.006** | **0.045** | **<0.001** | **0.001** | **0.002** | 0.157 |
| CRP | *ρ* |  | 0.205 | 0.107 | 0.107 | 0.176 | 0.097 | 0.267 | 0.044 |
|  | *p*-value |  | **<0.001** | **0.028** | **0.029** | **<0.001** | **0.047** | **<0.001** | 0.367 |
| Resistin | *ρ* |  |  | 0.623 | 0.665 | 0.575 | 0.542 | 0.425 | 0.232 |
|  | *p*-value |  |  | **<0.001** | **<0.001** | **<0.001** | **<0.001** | **<0.001** | **<0.001** |
| MPO | *ρ* |  |  |  | 0.695 | 0.624 | 0.552 | 0.329 | 0.150 |
|  | *p*-value |  |  |  | **<0.001** | **<0.001** | **<0.001** | **<0.001** | **0.001** |
| MMP-8 | *ρ* |  |  |  |  | 0.734 | 0.686 | 0.287 | 0.180 |
|  | *p*-value |  |  |  |  | **<0.001** | **<0.001** | **<0.001** | **<0.001** |
| MMP-9 | *ρ* |  |  |  |  |  | 0.682 | 0.348 | 0.133 |
|  | *p*-value |  |  |  |  |  | **<0.001** | **<0.001** | **0.003** |
| MMP-9/TIMP-1 complex | *ρ* |  |  |  |  |  |  | 0.272 | 0.173 |
|  | *p*-value |  |  |  |  |  |  | **<0.001** | **<0.001** |
| TIMP-1 | *ρ* |  |  |  |  |  |  |  | 0.406 |
|  | *p*-value |  |  |  |  |  |  |  | **<0.001** |

Comparisons were performed by Spearman’s Rank correlation

^*^WBC: white blood cells; ^†^CRP: C-reactive protein; ^‡^MPO: myeloperoxidase; ^§^MMP: matrix metalloproteinase; ^||^TIMP: tissue inhibitor of metalloproteinases.

# **Supplementary Table 4. Correlations between neutrophils activity biomarkers and clinical, anthropometric and laboratory characteristics.**

|  | CRP | | Resistin | | MPO | | MMP-8 | | MMP-9 | | MMP-9/TIMP-1 complex | | TIMP-1 | | TIMP-2 | |
| --- | --- | --- | --- | --- | --- | --- | --- | --- | --- | --- | --- | --- | --- | --- | --- | --- |
| Parameter | *ρ* | *p*-value | *ρ* | *p*-value | *ρ* | *p*-value | *ρ* | *p*-value | *ρ* | *p*-value | *ρ* | *p*-value | *ρ* | *p*-value | *ρ* | *p*-value |
| Age | -0.076 | 0.120 | 0.047 | 0.308 | -0.074 | 0.108 | -0.052 | 0.257 | -0.040 | 0.377 | -0.019 | 0.830 | 0.037 | 0.412 | 0.101 | 0.026 |
| Female sex | **-0.204** | **<0.001** | -0.020 | 0.656 | 0.066 | 0.146 | 0.065 | 0.154 | 0.045 | 0.314 | **0.108** | **0.016** | **-0.097** | **0.031** | 0.012 | 0.782 |
| Menopausal status | -0.109 | 0.183 | 0.004 | 0.959 | -0.061 | 0.419 | -0.050 | 0.513 | 0.007 | 0.923 | -0.017 | 0.822 | -0.021 | 0.783 | 0.039 | 0.608 |
| Duration of ^*^DM | 0.003 | 0.948 | **0.118** | **0.015** | -0.021 | 0.672 | 0.015 | 0.755 | 0.022 | 0.647 | 0.074 | 0.130 | 0.068 | 0.162 | 0.017 | 0.730 |
| Body weight | **0.204** | **<0.001** | -0.003 | 0.946 | 0.017 | 0.715 | 0.014 | 0.733 | 0.002 | 0.972 | 0.058 | 0.202 | 0.005 | 0.916 | -0.018 | 0.700 |
| ^†^BMI | **0.328** | **<0.001** | 0.042 | 0.363 | 0.034 | 0.464 | 0.030 | 0.514 | 0.033 | 0.465 | 0.033 | 0.471 | 0.078 | 0.089 | -0.014 | 0.760 |
| ^‡^WC | **0.278** | **<0.001** | 0.049 | 0.310 | 0.038 | 0.429 | 0.046 | 0.345 | 0.059 | 0.218 | 0.076 | 0.103 | 0.096 | 0.044 | 0.014 | 0.778 |
| Metabolic syndrome | **0.240** | **<0.001** | 0.079 | 0.080 | 0.045 | 0.325 | 0.022 | 0.301 | 0.047 | 0.301 | -0.002 | 0.972 | **0.187** | **<0.001** | 0.024 | 0.588 |
| Smoking habit | **-0.162** | **0.001** | 0.057 | 0.208 | 0.031 | 0.497 | 0.010 | 0.821 | -0.066 | 0.146 | 0.028 | 0.543 | -0.033 | 0.469 | 0.043 | 0.342 |
| ^\|\|^SBP | 0.068 | 0.166 | 0.073 | 0.110 | 0.041 | 0.372 | -0.005 | 0.914 | 0.008 | 0.852 | -0.083 | 0.068 | **0.139** | **0.002** | 0.045 | 0.318 |
| ^¶^DBP | **0.124** | **0.011** | **0.091** | **0.046** | **0.138** | **0.002** | 0.026 | 0.567 | 0.088 | 0.053 | 0.026 | 0.564 | **0.113** | **0.013** | 0.058 | 0.204 |
| Anti-hypertensive drugs | -0.005 | 0.926 | 0.085 | 0.070 | -0.048 | 0.308 | -0.022 | 0.634 | 0.004 | 0.926 | -0.062 | 0.183 | 0.062 | 0.188 | 0.007 | 0.878 |
| ^#^FBG | **0.099** | **0.043** | 0.011 | 0.801 | 0.010 | 0.828 | 0.025 | 0.580 | 0.028 | 0.534 | 0.042 | 0.353 | **0.102** | **0.024** | -0.044 | 0.330 |
| Fasting insulin | **0.233** | **<0.001** | 0.049 | 0.352 | 0.013 | 0.810 | 0.103 | 0.053 | -0.006 | 0.911 | 0.011 | 0.832 | **0.139** | **0.009** | 0.101 | 0.058 |
| ^**^HOMA-IR | **0.270** | **<0.001** | 0.036 | 0.502 | 0.030 | 0.568 | **0.108** | **0.042** | 0.007 | 0.895 | 0.030 | 0.570 | **0.166** | **0.002** | 0.057 | 0.283 |
| HOMA-β% | **0.125** | **0.024** | 0.030 | 0.572 | -0.038 | 0.472 | 0.040 | 0.457 | -0.029 | 0.585 | -0.050 | 0.346 | 0.056 | 0.289 | **0.122** | **0.021** |
| ^††^HbA1c | **0.223** | **<0.001** | 0.023 | 0.647 | 0.019 | 0.710 | 0.039 | 0.446 | 0.069 | 0.169 | 0.050 | 0.318 | **0.155** | **0.002** | -0.037 | 0.169 |
| Total-cholesterol | **0.173** | **<0.001** | 0.025 | 0.581 | 0.084 | 0.085 | **0.096** | **0.035** | 0.046 | 0.312 | 0.040 | 0.379 | -0.020 | 0.666 | -0.010 | 0.822 |
| Triglycerides | 0.095 | 0.050 | 0.087 | 0.054 | **0.096** | **0.033** | 0.082 | 0.071 | 0.065 | 0.154 | 0.078 | 0.086 | **-0.108** | **0.018** | -0.009 | 0.857 |
| ^‡‡^HDL-c | **-0.121** | **0.013** | -0.087 | 0.056 | -0.048 | 0.297 | 0.095 | 0.068 | -0.012 | 0.785 | -0.068 | 0.134 | **0.097** | **0.035** | -0.010 | 0.822 |
| ^§§^LDL-c | **0.197** | **<0.001** | 0.017 | 0.734 | 0.049 | 0.341 | 0.018 | 0.695 | 0.017 | 0.747 | 0.075 | 0.143 | -0.040 | 0.439 | 0.035 | 0.440 |

Comparisons were performed by Spearman’s Rank correlation.

^*^DM: diabetes mellitus; ^†^BMI: body mass index; ^‡^WC: waist circumference; ^||^SBP: systolic blood pressure; ^¶^DBP: diastolic blood pressure; ^#^FBG: fasting blood glucose; ^**^HOMA: homeostatic model of assessment for insulin resistance (IR) and beta-cell function (β%); ^††^HbA1c: glycated hemoglobin; ^‡‡^HDL-c: high-density lipoprotein cholesterol; ^§§^LDL-c: low-density lipoprotein cholesterol.

# **Supplementary Table 5. Overall significance of potential confounders.**

|  | **Normal** | **Restrictive/ Diastolic dysfunction** | **Dilative/ Systolic dysfunction** | **Overall *p*-value** |
| --- | --- | --- | --- | --- |
| Age, yrs | 56.0 [49.8-62.3] | 62.0 [55.0-70.0] | 62.5 [56.0-69.0] | **<0.001** |
| Duration of diabetes, yrs | 2 [0-9] | 3 [0-13] | 5 [1-14] | **0.047** |
| Sex, females, % | 31 (31.0) | 82 (43.6) | 50 (30.7) | **0.020** |
| Post-menopausal status, % | 13 (41.9) | 19 (23.2) | 15 (30.0) | 0.142 |
| Duration of menopause, yrs | 10 [6-22] | 18 [9-27] | 16 [7-22] | 0.368 |
| Body weight, kg | 88.4 [16.7] | 85.6 [20.9] | 83.2 [17.2] | 0.086 |
| ^*^BMI, kg/m^2^ | 31.5 [5.4] | 32.3 [6.9] | 30.7 [5.9] | 0.060 |
| Waist circumference, cm | 107.2 ± 11.3 | 108.6 ± 15.3 | 104.7 ± 13.4 | **0.046** |
| Systolic Blood Pressure, mmHg | 133.4 ± 15.5 | 136.7 ± 17.9 | 136.9 ± 18.7 | 0.232 |
| Diastolic Blood Pressure, mmHg | 80.8 ± 10.6 | 79.8 ± 10.8 | 78.4 ± 9.8 | 0.181 |
| Smoking habit, % |  |  |  |  |
| Non-smokers | 47 (47.0) | 101 (53.7) | 64 (39.5) | **0.031** |
| Former smokers | 23 (23.0) | 28 (14.9) | 28 (17.3) |  |
| Current smokers | 30 (30.0) | 59 (31.4) | 70 (43.2) |  |
| Hypertension, % | 77 (79.4) | 168 (89.8) | 149 (94.3) | **0.001** |
| Anti-hypertensive drugs, |  |  |  |  |
| 0 | 30 (31.9) | 43 (23.8) | 22 (15.1) | **0.013** |
| 1 | 27 (28.7) | 49 (27.1) | 31 (21.2) |  |
| 2 | 20 (21.3) | 46 (25.4) | 48 (32.9) |  |
| 3 or more | 17 (18.1) | 43 (23.8) | 45 (30.8) |  |
| Dyslipidemia, % | 35 (37.2) | 73 (40.3) | 79 (54.1) | **0.013** |
| Lipid lowering drugs |  |  |  |  |
| No/diet, n (%) | 68 (64.2) | 100 (58.8) | 67 (45.9) | 0.090 |
| Statin, n (%) | 38 (35.8) | 68 (40.0) | 76 (52.1) |  |
| Fibrate, n (%) | 0 (0.0) | 1 (0.6) | 2 (1.4) |  |
| Statin + fibrate, n (%) | 0 (0.0) | 1 (0.6) | 1 (0.7) |  |
| Metabolic Syndrome, % | 76 (76.0) | 155 (82.4) | 120 (73.6) | 0.123 |
| White blood cells, x10^3^/mL | 7.2 [5.9-8.3] | 7.1 [6.0-8.8] | 7.5 [6.3-8.8] | 0.159 |
| Neutrophils, x10^3^/mL | 3.7 [2.6-4.7] | 4.3 [3.3-4.9] | 4.4 [3.7-5.3] | 0.324 |
| Fasting Blood Glucose, mg/dL | 131 [107-154] | 136 [117-175] | 136 [111-174] | 0.161 |
| Fasting Insulin, UI/mL | 14.0 [10.9-23.1] | 14.0 [10.0-21.7] | 13.0 [8.5-23.4] | 0.456 |
| ^†^HOMA-IR | 4.8 [3.3-7.8] | 4.8 [3.2-8.0] | 4.5 [2.3-7.3] | 0.374 |
| HOMA-β% | 90.0 [44.4-163.2] | 77.5 [42.6-128.8] | 78.1 [38.4-134.2] | 0.506 |
| ^‡^HbA1c, % | 6.5 [6.0-7.7] | 6.9 [6.2-7.8] | 7.1 [6.2-9.2] | **0.037** |
| Total cholesterol, mg/dL | 184 [163-218] | 191 [156-224] | 179 [148-216] | 0.057 |
| Triglycerides, mg/dL | 145 [109-180] | 134 [105-191] | 137 [101-190] | 0.941 |
| ^§^HDL-cholesterol, mg/dL | 43 [36-51] | 46 [37-55] | 43 [36-52] | 0.126 |
| ^\|\|^LDL-cholesterol, mg/dL | 111 [87-145] | 115 [84-145] | 108 [77-137] | 0.169 |

Continuous variables are presented and median [IQR] or mean ± SD according with their distribution; categorical ones are presented as absolute count (%). Comparisons were then drawn by one-way ANOVA, Kruskal-Wallis test and χ^2^ test, as appropriate.

^*^BMI: body mass index; ^†^HOMA: homeostatic model of assessment for insulin resistance (IR) and beta-cell function (β%); ^‡^HbA1c: glycated hemoglobin; ^§^HDL: high-density lipoprotein; ^||^LDL: low-density lipoprotein.

# **Supplementary Table 6. Pairwise comparisons by univariate logistic analysis.**

|  | **Restrictive/diastolic dysfunction vs. normal pattern** | | **Dilative/systolic dysfunction vs. normal** | | **Restrictive/diastolic vs. dilative/systolic dysfunction** | |
| --- | --- | --- | --- | --- | --- | --- |
| **Parameter** | ^*^**OR (95% CI)** | ***p*-value** | **OR (95% CI)** | ***p*-value** | **OR (95% CI)** | ***p*-value** |
| Age | **1.06 (1.03-1.09)** | **<0.001** | **1.08 (1.05-1.11)** | **<0.001** | 1.01 (0.99-1.03) | 0.481 |
| Duration of diabetes >4 yrs | 1.23 (0.72-2.11) | 0.451 | 1.71 (0.98-2.97) | 0.060 | 1.39 (0.88-2.18) | 0.157 |
| Female sex | **1.72 (1.03-1.88)** | **0.038** | 0.99 (0.58-1.69) | 0.956 | **0.57 (0.37-0.89)** | **0.013** |
| Waist circumference | 1.01 (0.99-1.03) | 0.431 | 0.99 (0.97-1.01) | 0.155 | **0.98 (0.97-1.00)** | **0.020** |
| Smoking habit |  | 0.228 |  | 0.093 |  | **0.025** |
| Former smokers (ref=never) | 0.57 (0.30-1.09) | 0.087 | 0.89 (0.46-1.74) | 0.742 | 1.58 (0.86-2.91) | 0.143 |
| Current smokers (ref=never) | 0.92 (0.52-1.60) | 0.756 | 1.71 (0.97-3.03) | 0.064 | **1.87 (1.17-2.99)** | **0.008** |
| Hypertension | **2.30 (1.16-4.55)** | **0.017** | **4.30 (1.87-9.90)** | **0.001** | 1.87 (0.82-4.26) | 0.135 |
| Anti-hypertensive drugs |  | 0.398 |  | **0.002** |  | 0.062 |
| 1 (ref=no) | 1.27 (0.65-2.45) | 0.485 | 1.57 (0.74-3.33) | 0.244 | 1.24 (0.63-2.45) | 0.542 |
| 2 (ref=no) | 1.61 (0.80-3.24) | 0.187 | **3.27 (1.53-6.99)** | **0.002** | **2.04 (1.06-3.92)** | **0.033** |
| 3 or more (ref=no) | 1.77 (0.85-3.67) | 0.127 | **3.61 (1.65-7.90)** | **0.001** | **2.05 (1.06-3.97)** | **0.034** |
| Dyslipidemia | 1.14 (0.68-1.90) | 0.618 | **1.99 (1.17-3.38)** | **0.011** | **1.74 (1.12-2.71)** | **0.013** |
| ^‡^HbA1c (%) | 1.26 (0.33-4.83) | 0.734 | **3.59 (1.03-12.56)** | **0.046** | **3.53 (1.14-10.95)** | **0.029** |

^*^OR: odds ratio; ^†^CI: confidence interval; ^‡^HbA1c: glycated haemoglobin.

# **Supplementary Table 7. Multivariate logistic regression analysis.**

|  | **Unadjusted** | | **Adjusted** | |
| --- | --- | --- | --- | --- |
| **Restrictive/diastolic dysfunction vs. normal pattern** | ^*^**OR (95% CI)** | ***p*-value** | **OR (95% CI)** | ***p*-value** |
| Resistin | **1.68 (1.22-2.32)** | **0.001** | **1.79 (1.27-2.52)** | **0.001** |
| Age | **1.06 (1.03-1.09)** | **<0.001** | **1.06 (1.03-1.09)** | **<0.001** |
| Female sex | **1.72 (1.03-2.88)** | **0.038** | 1.30 (0.74-2.28) | 0.365 |
| Hypertension | **2.30 (1.16-4.55)** | **0.017** | 1.74 (0.82-3.70) | 0.148 |
|  |  |  |  |  |
| ^‡^MPO | **1.37 (1.07-1.75)** | **0.014** | **1.57 (1.20-2.06)** | **0.001** |
| Age | **1.06 (1.03-1.09)** | **<0.001** | **1.07 (1.04-1.10)** | **<0.001** |
| Female sex | **1.72 (1.03-1.88)** | **0.038** | 1.44 (0.81-2.53) | 0.212 |
| Hypertension | **2.30 (1.16-4.55)** | **0.017** | 1.73 (0.82-3.68) | 0.152 |
|  |  |  |  |  |
| ^§^ MMP-8 | **1.28 (1.01-1.64 )** | **0.044** | **1.39 (1.06-1.81)** | **0.016** |
| Age | **1.06 (1.03-1.09)** | **<0.001** | **1.06 (1.03-1.09)** | **<0.001** |
| Female sex | **1.72 (1.03-1.88)** | **0.038** | 1.39 (0.79-2.44) | 0.258 |
| Hypertension | **2.30 (1.16-4.55)** | **0.017** | 1.73 (0.82-3.67) | 0.151 |
|  |  |  |  |  |
| MMP-9/^¶^TIMP-1 complex | **1.36 (1.07-1.74)** | **0.014** | **1.47 (1.12-1.91)** | **0.005** |
| Age | **1.06 (1.03-1.09)** | **<0.001** | **1.06 (1.03-1.09)** | **<0.001** |
| Female sex | **1.72 (1.03-1.88)** | **0.038** | 1.46 (0.83-2.57) | 0.190 |
| Hypertension | **2.30 (1.16-4.55)** | **0.017** | 1.80 (0.85-3.82) | 0.126 |
|  |  |  |  |  |
| TIMP-1 | **2.27 (1.09-4.72)** | **0.028** | 2.09 (0.96-4.52) | 0.063 |
| Age | **1.06 (1.03-1.09)** | **<0.001** | **1.06 (1.03-1.09)** | **<0.001** |
| Female sex | **1.72 (1.03-1.88)** | **0.038** | 1.29 (0.74-2.26) | 0.375 |
| Hypertension | **2.30 (1.16-4.55)** | **0.017** | 1.54 (0.73-3.25) | 0.255 |
|  |  |  |  |  |
| TIMP-2 | **2.31 (1.08-4.91)** | **0.030** | 1.64 (0.74-3.64) | 0.220 |
| Age | **1.06 (1.03-1.09)** | **<0.001** | **1.06 (1.03-1.08)** | **<0.001** |
| Female sex | **1.72 (1.03-1.88)** | **0.038** | 1.35 (0.78-2.36) | 0.285 |
| Hypertension | **2.30 (1.16-4.55)** | **0.017** | 1.61 (0.76-3.37) | 0.211 |

^*^OR: odds ratio; ^†^CI: confidence interval; ^‡^MPO: myeloperoxidase; ^§^ MMP: matrix metalloproteinase; ^¶^TIMP: tissue inhibitor of metalloproteinase

# **Supplementary Table 8. Multivariate logistic regression analysis.**

|  | **Unadjusted** | | **Adjusted** | |
| --- | --- | --- | --- | --- |
| **Dilative/systolic dysfunction pattern vs restrictive/ diastolic dysfunction pattern** | ^*^**OR (95%** ^†^**CI)** | ***p*-value** | **OR (95% CI)** | ***p*-value** |
| Resistin | **0.70 (0.50-0.99)** | **0.046** | **0.67 (0.46-0.99)** | **0.042** |
| Female sex | **0.57 (0.37-0.39)** | **0.013** | **0.50 (0.26-0.95)** | **0.035** |
| Waist circumference (cm) | **0.98 (0.97-1.00)** | **0.020** | **0.98 (0.96-1.00)** | **0.026** |
| Smoking habit |  | **0.025** |  | 0.578 |
| Former smokers (ref=never) | 1.58 (0.86-2.91) | 0.143 | 1.51 (0.61-3.71) | 0.371 |
| Current smokers (ref=never) | **1.87 (1.17-2.99)** | **0.008** | 1.34 (0.70-2.58) | 0.380 |
| Anti-hypertensive drugs |  | 0.062 |  | 0.089 |
| 1 (ref=no) | 1.24 (0.63-2.45) | 0.542 | 1.14 (0.48-2.69) | 0.768 |
| 2 (ref=no) | **2.04 (1.06-3.92)** | **0.033** | 2.14(0.93-4.92) | 0.073 |
| 3 or more (ref=no) | **2.05(1.06-3.97)** | **0.034** | **2.46 (1.03-5.92)** | **0.044** |
| Dyslipidemia | **1.74 (1.12-2.71)** | **0.013** | 1.12 (0.63-1.99) | 0.693 |
| ^‡^HbA1c (%) | **3.53 (1.14-10.95)** | **0.029** | **6.46 (1.70-24.55)** | **0.006** |

^*^OR: odds ratio; ^†^CI: confidence interval; ^‡^HbA1c: glycated haemoglobin.

# **Supplementary Table 9. Multivariate logistic regression analysis.**

|  | **Unadjusted** | | **Adjusted** | |
| --- | --- | --- | --- | --- |
| **Dilative/ systolic dysfunction pattern vs normal pattern** | ^*^**OR (95%** ^†^**CI)** | ***p*-value** | **OR (95% CI)** | ***p*-value** |
| ^‡^MPO | 1.30 (0.97-1.76) | 0.084 | **1.58 (1.10-2.52)** | **0.013** |
| Age | **1.08 (1.05-1.11)** | **<0.001** | **1.07 (1.04-1.11)** | **<0.001** |
| Hypertension | **2.30 (1.16-4.55)** | **0.017** | 2.44 (0.63-9.47) | 0.196 |
| Anti-hypertensive drugs |  | **0.002** |  | 0.284 |
| 1 (ref=no) | 1.57 (0.74-3.33) | 0.244 | 1.08 (0.35-3.32) | 0.899 |
| 2 (ref=no) | **3.27 (1.53-6.99)** | **0.002** | 1.99 (0.61-6.52) | 0.254 |
| 3 or more (ref=no) | **3.61 (1.65-7.90)** | **0.001** | 2.48 (0.71-8.62) | 0.154 |
| Dyslipidemia | **1.99 (1.17-3.38)** | **0.011** | 1.20 (0.59-2.45) | 0.616 |
| ^§^HbA1c (%) | **3.59** (**1.03-12.56**) | **0.046** | **5.02 (1.11-22.64)** | **0.036** |

^*^OR: odds ratio; ^†^CI: confidence interval; ^‡^MPO: myeloperoxidase ^§^HbA1c: glycated haemoglobin.


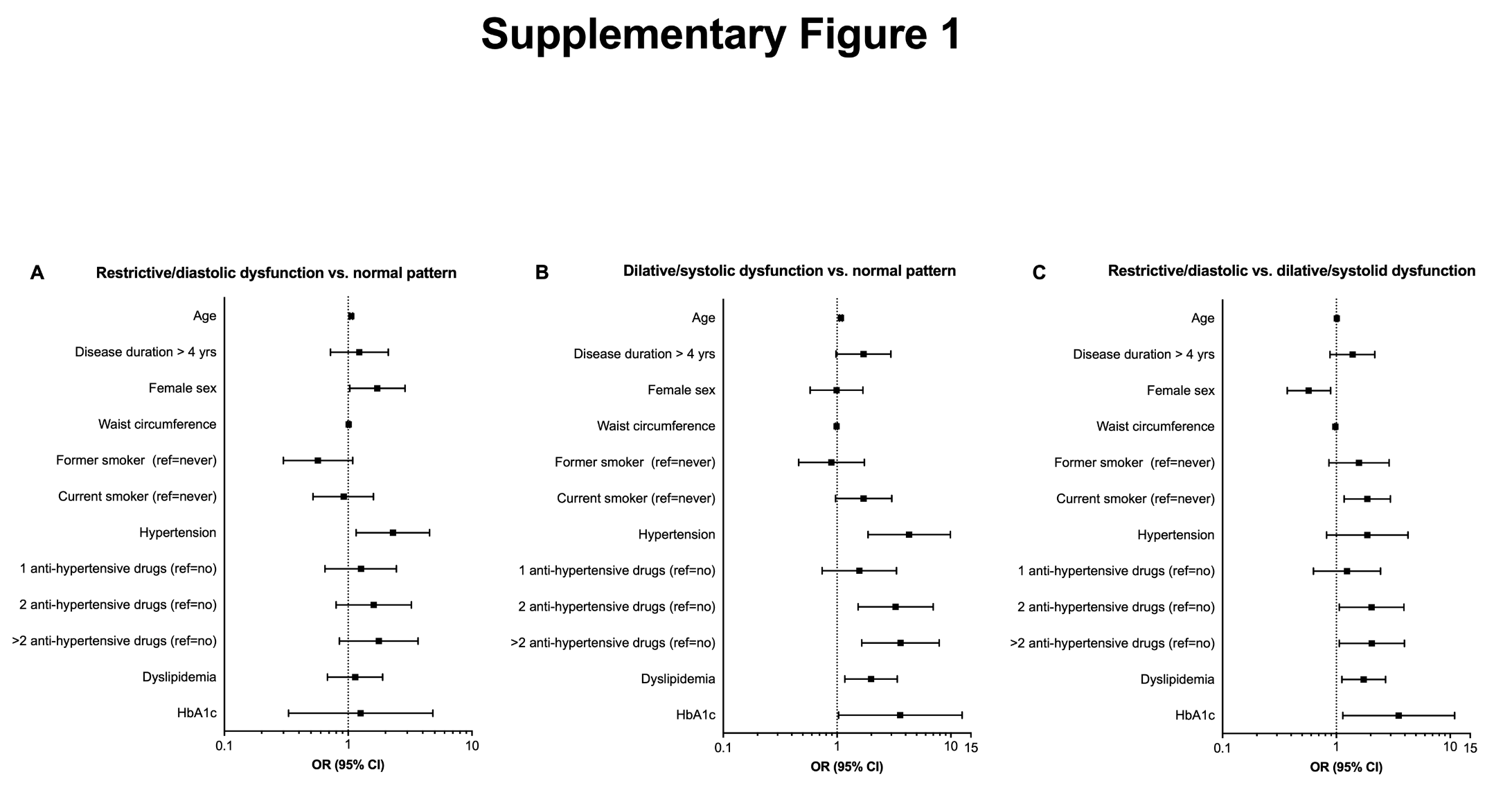


# **Supplementary Figure 1. Logistic regression analyses.**

Logistic regression analyses were performed to investigate clinical variables independently associated with restrictive/diastolic dysfunction **(A)** and dilative/systolic dysfunction **(B)** as compared with normal echocardiographic pattern. Panel **C** also shown potential determinants differentiating the development of restrictive/diastolic vs. dilative/systolic left ventricular dysfunction.
